# Cryo-EM structure of human SAGA transcriptional coactivator complex

**DOI:** 10.1038/s41421-022-00489-w

**Published:** 2022-11-22

**Authors:** Yuzhu Zhang, Changping Yin, Yue Yin, Mengqi Wei, Wei Jing, Chao Peng, Zhengjun Chen, Jing Huang

**Affiliations:** 1 Shanghai Key Laboratory of Stomatology, Shanghai Ninth People’s Hospital, College of Stomatology, Shanghai Jiao Tong University School of Medicine, Shanghai, China; 2 Shanghai Institute of Precision Medicine, Shanghai, China; 3 State Key Laboratory of Molecular Biology, CAS Center for Excellence in Molecular Cell Science, Shanghai Institute of Biochemistry and Cell Biology, University of Chinese Academy of Sciences, Chinese Academy of Sciences, Shanghai, China; 4 National Facility for Protein Science in Shanghai, Zhangjiang Lab, Shanghai Advanced Research Institute, Chinese Academy of Science, Shanghai, China; 5 School of Life Sciences and Biotechnology, Shanghai Jiao Tong University, Shanghai, China

**Keywords:** Cryoelectron microscopy, Epigenetics

Dear Editor,

Transcription initiation is an essential regulatory step of gene expression that requires coordinated functions of transcription factors and chromatin regulators to provide accessible chromatin environment for the assembly of transcription pre-initiation complex. In this process, the transcription regulatory proteins are often organized into a number of modular and multi-functional coactivator complexes to orchestrate their different activities. Evolutionarily conserved with yeast SAGA (Spt–Ada–Gcn5 acetyltransferase) complex, human SAGA (abbreviated as hSAGA) complex is a well-established transcription coactivator that regulates the transcription of numerous genes^1^. Several distinct functional modules have been characterized for the 1.4-MDa SAGA complex, including a histone acetyltransferase (HAT) module and a histone deubiquitinase (DUB) module that establish histone modification signatures for transcription activation, the largest subunit TRRAP that directly interacts with transcription activators, the core module that serves as a structural scaffold, and the spliceosome U2 snRNP factors SF3B3 and SF3B5^1,2^ (Supplementary Fig. S1). Recent cryo-electron microscopy (cryo-EM) structures of yeast SAGA complex elucidated the structural organizations of the SAGA modules and the mechanism of TATA box-binding protein (TBP) recruitment and delivery for the nucleation of pre-initiation complex^3,4^. However, TBP was barely identified in the interactome of hSAGA complex^5,6^, and the spliceosome factors SF3B3 and SF3B5 are constantly incorporated into hSAGA complex^1^. The organization and functional mechanisms of hSAGA complex remain poorly understood.

Here we report the cryo-EM structure of endogenous hSAGA coactivator complex purified from HEK293 cells at an overall resolution of 3.7 Å (Supplementary Figs. S2, S3 and Table S1). Focused refinement on TRRAP improved its resolution to 3.4 Å and masked 3D classifications without realignment were applied to improve the local EM densities of the core module and the spliceosome factors (Supplementary Fig. S2a). Most of the HAT and DUB modules were invisible in the final EM map of hSAGA, suggesting that their attachment is largely flexible. Homology modeling and de novo chain building were used to generate an atomic model of the hSAGA complex (Fig. 1a, b and Supplementary Table S2). Cross-linking mass spectrometry analysis was performed to guide model building (Supplementary Fig. S1d and Tables S3, S4).

**Fig. 1:**
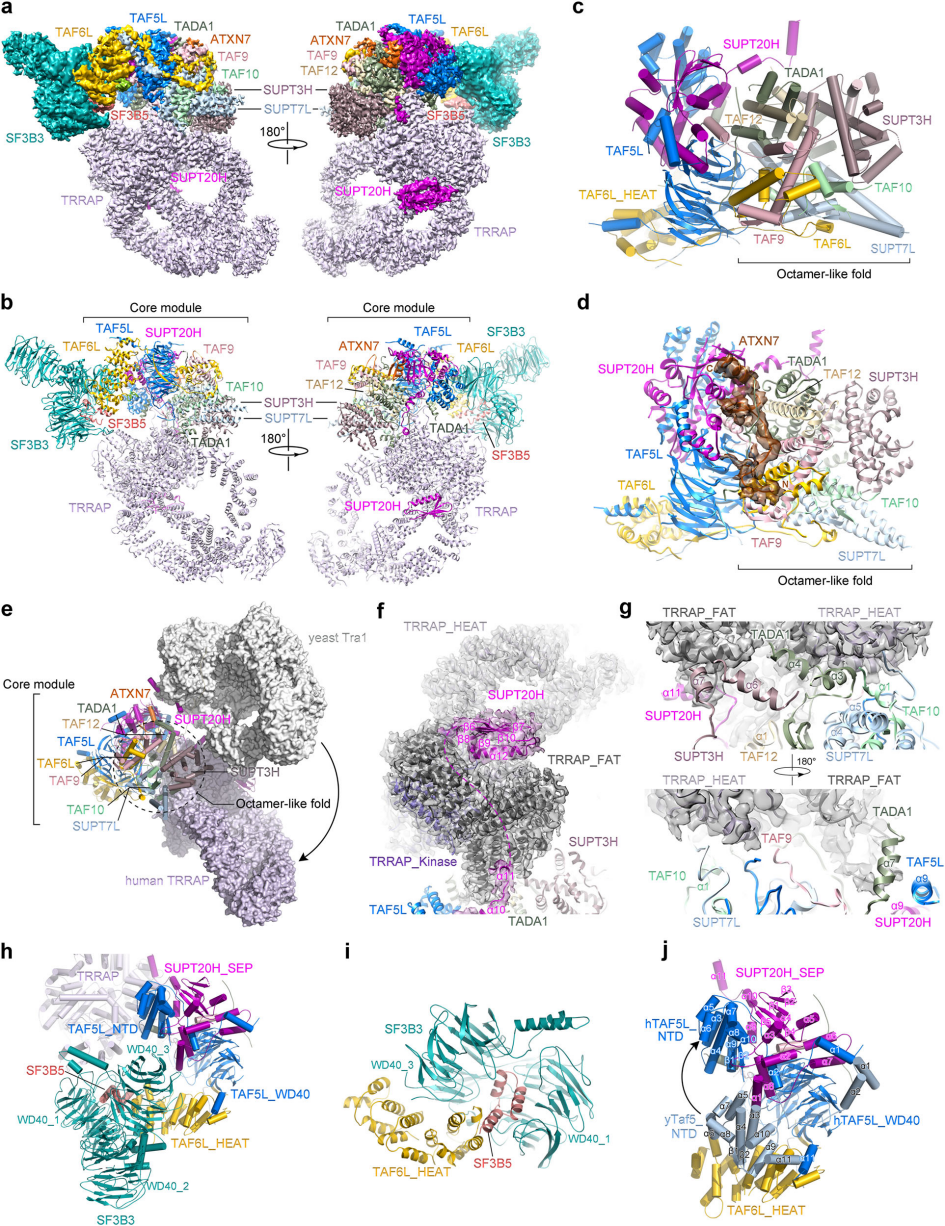
Structural characterization of human SAGA complex. **a**, **b** Cryo-EM density map (**a**) and atomic model (**b**) of hSAGA complex are shown in front and back views. The cryo-EM map was segmented and colored according to different subunits of hSAGA complex and was shown at a contour level of 0.015 (SF3B3/5 was shown at a contour level of 0.009). **c** Overview of the core module. The histone octamer-like fold and the remainder structures are denoted, respectively. **d** Overall structural organization of the interface between ATXN7 and the core module. The ATXN7 segment is shown with EM density maps at a contour level of 0.015. **e** Structural comparison of hSAGA complex and yeast SAGA complex shows different binding ways of TRRAP and Tra1 to the core modules. **f** Overview of the binding path of SUPT20H on the surface of TRRAP. **g** Interface between TRRAP and the core module components, shown in front and back views. **h** Overall organization of the SF3B3/5 module within the complex. **i** Interface among SF3B3, SF3B5, and the HEAT repeat domain of TAF6L. **j** Structural superposition of the core modules of hSAGA complex and yeast SAGA complex reveals different organizations of the N-terminal domains of human TAF5L and yeast Taf5 in the two complexes.

Human SAGA coactivator forms a rhomboid architecture, with the core module serving as a central hub for the assembly of TRRAP, SF3B3/5 and the DUB module into the complex (Fig. 1a, b and Supplementary Fig. S4a). The core module contains nine highly interconnected protein components, among which the histone-fold domains of TAF6L, TAF9, TAF10, TAF12, SUPT3H, SUPT7L, and TADA1 are assembled into an asymmetric histone octamer-like fold (Fig. 1c). This structural feature is evolutionarily conserved in yeast SAGA complex and is also found in general transcription factor IID that shares a subset of TBP-associated factors (TAFs) with hSAGA^3,4^ (Supplementary Fig. S4b). The remainder of the core module, including TAF5L, SUPT20H and the HEAT repeat domain of TAF6L, exhibits an extended and curved organization and intimately contacts the octamer-like region (Fig. 1c and Supplementary Fig. S4c). In the vicinity of the interface between SUPT20H and the octamer-like region, residues 509–552 of ATXN7 from the DUB module meanders through the core module and extends toward an extra EM density that is visible in low-resolution maps of hSAGA (Fig. 1d and Supplementary Figs. S4a, S5a). Comparison with yeast SAGA structure revealed that Sgf73, the yeast homolog of ATXN7, occupies the similar position as the ATXN7 fragment does in the core module (Supplementary Fig. S5b). In addition, structural superposition of the core modules of yeast SAGA and hSAGA complexes could fit the yeast DUB module exactly in the extra density map of hSAGA, suggesting that the DUB module is connected to hSAGA complex in a similar way as that in yeast SAGA complex (Supplementary Fig. S4a). HAT module is attached to the HEAT repeat of yeast Taf6 through a few helices of Ada3 in yeast SAGA complex^3^. Over the same surface of human TAF6L, an unassigned EM density was found at the similar position where yeast Ada3 is located, suggesting that the unassigned chain might be TADA3 from the HAT module of hSAGA (Supplementary Fig. S5c, d).

Human TRRAP is attached to the core module in a remarkably different way from its yeast counterpart Tra1 (Fig. 1e). In contrast with the loose connection of Tra1 to the core module of yeast SAGA, the TRRAP module comes close to the octamer-like region of the core module and makes extensive contacts with the core module components, with a buried surface area of 5000 Å^2^ (Fig. 1e). TRRAP is primarily tethered by discrete fragments of SUPT20H (Fig. 1f). Helix α11 of SUPT20H passes through a groove of the FAT domain of TRRAP, and then a C-terminal αβ structure of SUPT20H is snugly nested at the joint region of the three TRRAP domains, strengthening the SUPT20H–TRRAP association (Fig. 1f and Supplementary Fig. S6a). Furthermore, at the interface between TRRAP and the octamer-like region of the core module, the helices α3 and α4 of TADA1 join the helix bundles of the HEAT repeats and FAT domains of TRRAP, and multiple loop and helical regions from SUPT3H, SUPT7L, TADA1, TAF9, TAF10 and TAF12 also contact TRRAP to coordinate the association (Fig. 1g and Supplementary Fig. S6b). The different organization of TRRAP relative to the core module confers a unique architecture of hSAGA complex compared with its yeast counterpart. Previous structural analysis suggested that Tra1 sterically obstructs the path of the TBP-bound DNA in yeast SAGA complex^3^. Compared with the organization of Tra1 and Spt3 in yeast SAGA complex, human TRRAP is located away from the TBP-binding pocket of SUPT3H and no longer hinders the path of the TBP-bound DNA (Supplementary Fig. S7a). In yeast SAGA, TBP is recruited to the complex through coordinated interactions with both Spt3 and Spt8^7^. The conserved TBP-binding pocket of SUPT3H (human orthologue of yeast Spt3) is vacant and potentially available for the association of TBP (Supplementary Fig. S7b), but an Spt8 homolog has yet to be identified in human cells. The master transcription factor c-MYC is a well-characterized binding partner of hSAGA complex and it also recruits TBP through cooperative interactions with the transcription initiation factor TAF1^8^, which provides a clue about the potential dynamic recruitment of TBP through the simultaneous interactions of c-MYC with both TBP and the hSAGA complex. It remains to be explored whether TBP could be incorporated into the functions of hSAGA complex through c-MYC-mediated interactions.

The splicing factors SF3B3 and SF3B5 are associated with the core module of hSAGA complex through the concave surface of the HEAT repeat domain of TAF6L (Fig. 1h and Supplementary Fig. S6c). The three-helix bundle of SF3B5 connects to the helix bundles of the HEAT repeat domain of TAF6L, which jointly cradles the third WD40 domain of SF3B3 (Fig. 1i). Structural comparison with yeast SAGA complex reveals that the concave surface of the HEAT repeat domain of yeast Taf6 is occupied by the N-terminal domain of yeast Taf5, which precludes the recruitment of the splicing factors to yeast SAGA complex; while in hSAGA complex, the N-terminal domain of human TAF5L gets away from the HEAT repeat domain of TAF6L and is tightly associated with the N-terminal region of SUPT20H, which allows the attachment of SF3B3 and SF3B5 to TAF6L (Fig. 1j). The repositioning of the N-terminal domain of TAF5L is possibly related to the gene duplication of yeast Taf5 and the subsequent sub-functionalization of the hSAGA-specific TAF5L and the TFIID-specific TAF5. The interfaces between the N-terminal domain of TAF5L and the N-terminal region of SUPT20H are highly conserved in metazoan species, but show poor sequence conservation with their yeast counterparts, suggesting that the interaction between the N-terminal domains of TAF5L and SUPT20H co-evolved during the evolutionary process of *Taf5* divergence (Supplementary Figs. S8, S9). Consistent with this, SF3B3 and SF3B5 have also been identified within the *Drosophila* SAGA complex.

SF3B3 and SF3B5 usually serve as the structural components of the SF3B complex in the spliceosome U2 snRNP. Structural superposition with the SF3B complex revealed that TAF6L occupies the surface where the HEAT repeat domain of SF3B1 interacts with SF3B3 and SF3B5, suggesting that the incorporation of SF3B3 and SF3B5 into the two complexes is mutually exclusive (Supplementary Fig. S10). Although SF3B3 and SF3B5 are absent from yeast SAGA complex, it has been shown that yeast U2 snRNP proteins Lea1 and Msl1 exhibit functional interactions with multiple subunits of the SAGA complex, particularly with Gcn5, whose HAT activity facilitates co-transcriptional recruitment of U2 snRNP to the branch points of pre-mRNA^9^. Moreover, the TBP-binding module (Spt3 and Spt8) of the yeast SAGA complex can recruit the U2 snRNP ATPase Prp5 to the promoter region, and consequently mediating a balance between transcription and pre-spliceosome assembly^10^. Accordingly, we presume that the incorporation of SF3B3 and SF3B5 into hSAGA complex might play a similar role in the coordination of transcription initiation and pre-mRNA splicing. Further studies will be required to examine the specific roles of SF3B3 and SF3B5 within the context of hSAGA complex by generating mutations that selectively disrupt the attachment of SF3B3 and SF3B5 to TAF6L.

In summary, our cryo-EM analysis reveals that human SAGA complex adopts an overall architecture distinct from yeast SAGA complex due to the different organization of TRRAP relative to the core module (Supplementary Fig. S11). TRRAP directly binds to several multifunctional transcription factors such as c-MYC, E2F and P53, and serves as an essential cofactor for the related transcription regulation^2^, further indicating the possible functional differences between human and yeast SAGA in multiple physiological activities. Future work on the structural mechanism for the regulation of hSAGA on transcription factors and its substrate nucleosomes will help understand how the unique configuration of hSAGA contributes to its functions. Our studies also elucidate the structural basis for the association of the splicing factors SF3B3 and SF3B5 with hSAGA complex and will promote functional investigations on the roles of the splicing factors in hSAGA complex.

## Supplementary information

Supplementary Information

Supplementary Table S3

Supplementary Table S4

## Data Availability

The cryo-EM structure and density map of hSAGA complex have been deposited at the Protein Data Bank and Electron Microscopy Data Bank under the accession codes of 8H7G and EMD-34520, respectively.
