# Supplementary material for: Cryo-EM structure of human SAGA transcriptional coactivator complex
Source: Cell Discov. 2022 Nov 22;8:125. doi: 10.1038/s41421-022-00489-w (PMC9681738; doi:10.1038/s41421-022-00489-w)
Supplement: Supplementary file 1 — Supplementary Information [file 41421_2022_489_MOESM1_ESM.pdf]

Supplementary information for

# Cryo-EM structure of human SAGA transcriptional coactivator complex

**Authors:** Yuzhu Zhang[1,2], Changping Yin[3], Yue Yin[4], Mengqi Wei[3], Wei Jing[3], Chao Peng[4], Zhengjun Chen[3], Jing Huang[1,2,5]*

Correspondence to: huangjing@shsmu.edu.cn

**This file includes:**

Materials and Methods
Supplementary References
Figures S1 to S11
Table S1 to S2

## Materials and Methods

### Stable cell line establishment

To prepare hSAGA complex for structural determination, a stable HEK293 cell line that overexpressed TADA1 and SUPT7L, two of the hSAGA subunits, was established using lentivirus transfection systems. TADA1 was cloned into a modified lentivirus expression vector pLVX-IRES-mCherry vector with an N-terminal 3xFlag tag, and SUPT7L was inserted into a modified lentivirus expression vector pLVX-IRES-EGFP vector with a C-terminal twin-Strep tag. The constructed lentiviral expression vectors together with the virus packaging plasmids were introduced into HEK293T cells using x-tremeGENE HP transfection reagent (Roche) according to the transfection protocol. The virus-containing supernatants derived from the HEK293T cells were collected at 36 hours post-transfection and were used to infect Expi293F cells (Thermo Fisher). The $EGFP^{+}$/$mCherry^{+}$ cells were sorted out by flow cytometry at 72 hours post-infection. The HEK293T cells were cultured in DMEM mediums supplemented with 10% FBS, and the Expi293F cells were cultured in CD 293 mediums (Cell-Wise).

### Purification of human SAGA complex

Approximately 12 L of the Expi293F cells overexpressed with TADA1 and SUPT7L proteins were harvested by centrifugation, washed in PBS and were then resuspended in lysis buffer (50 mM HEPES pH 7.5, 150 mM NaCl, 12 mM $MgCl_2$, 10% glycerol, 0.04% NP-40, 5 mM 2-mercaptoethanol, 1 mM PMSF, 5 mM benzamidine, 1 μg/ml leupeptin, and 1 μg/ml pepstatin). The suspended cells were frozen to 3~5 mm beads by liquid nitrogen and the cell beads were broken down by SPEX6870D Freezer Mill. The cell lysates were cleared by centrifugation at 19,500 rpm for 1 hour at 4 ºC and the supernatant was then bound with anti-Flag G1 affinity resin (GenScript) for 12 hours. The resin was washed with wash buffer (50 mM HEPES pH 7.5,

150 mM NaCl, 12 mM $MgCl_2$, 10% glycerol, 5 mM 2-mercaptoethanol, 1 mM PMSF) and the protein complex was eluted in wash buffer supplemented with 500 ng/μl Flag peptides (WHSTbio). The eluent was then incubated with Strep-Tactin XT Superflow high-capacity resin (IBA Lifesciences) overnight. The resin was washed extensively and the complex was eluted in wash buffer containing 50 mM D-biotin (YEASEN). The hSAGA complex was concentrated to about 0.6 mg/ml and was used immediately.

**Sample preparation and cryo-EM data collection**

Purified hSAGA complex was incubated with 0.1% glutaraldehyde for 20 minutes on ice and was then purified using the method of GraFix[1]. In brief, a continuous 10-50% gradient of glycerol with a 0-0.1% gradient of glutaraldehyde was generated on a BioComp gradient master. Then the hSAGA complex was loaded on top of the glycerol gradient in the buffer of 20 mM HEPES pH 7.5, 150 mM NaCl, 5 mM $MgCl_2$ and 1 mM tris (2-carboxyethyl) phosphine (TCEP), and the ultracentrifugation was performed at the speed of 37,000 rpm at 4 ºC for 15 hours using a SW 60 Ti rotor (Beckman). The Grafix fractions containing the hSAGA complex was collected and the cross-linking was quenched by 50 mM Tris-HCl pH 7.5. The samples were then dialyzed against 25 mM HEPES pH 7.5, 150 mM NaCl and 1 mM DTT, and were concentrated to about 0.4 mg/ml. 2 μl aliquots of the samples were applied to lacey-carbon grids with an ultra-thin carbon film (Ted Pella) preprocessed with glow-discharging at 0.42 mbar and 25 mA for 25 s, and the grids were blotted for 3 s and were then plunged into liquid ethane cooled by liquid nitrogen using a Vitrobot Mark IV (FEI).

Cryo-EM images were acquired on a Titan Krios transmission electron microscope (FEI) operated at 300 kV. The images were collected on a K3 direct electron detector (Gatan) with a pixel size of 1.10 Å. Automated image acquisition was performed with EPU software (FEI). The

dataset was collected at a defocus varying between -0.8 to -3.0 µm, and each micrograph was dose-fractioned to 32 frames with 0.1-s exposure time for each frame. The total accumulated dose of each micrograph is 50.0 $e^-/Å^2$. The imaging conditions were also listed in Table S1.

**Image processing and model building**

A total of 5,036 cryo-EM images of hSAGA complex were collected on a K3 detector, and motion correction was performed on the dose-fractioned image stacks using MotionCor2 with dose weighting[2,3]. The contrast-transfer function (CTF) parameters of each image were determined with Gctf[4]. Particle picking, 2D classification, 3D initial model, 3D classification, 3D auto-refine, CTF refinement, and Bayesian polishing were performed with RELION-3.1[5]. We manually picked about 1000 particles to generate the templates for particle auto-picking using Gautomatch (http://www.mrc-lmb.cam.ac.uk/kzhang/Gautomatch/) for further processing. An overview of the data processing procedure was shown in Supplementary Fig. S2a. After two rounds of 2D classification and two rounds of 3D classification, a total of 378,168 particles that belong to the hSAGA complex were processed with 3D auto-refinement and solvent-masked post-processing, which generated a cryo-EM density map with an overall resolution of 3.7 Å. To improve the map densities of TRRAP, the core module, and the SF3B3/5 module, the particles were further processed through focused 3D refinement and masked 3D classifications with partial signal subtraction, respectively[6]. The resolution estimation was based on the gold-standard Fourier shell correlation (FSC) 0.143 criterion and the local resolution was estimated with ResMap[7,8].

Model building was carried out by fitting the available structures of yeast SAGA (PDB codes: 6T9I, 6TBM and 6MZD) in the electron microscopy density map of the hSAGA complex

using UCSF Chimera[9]. The model was then manually built in Coot and real-space-refined with secondary structure restraints in Phenix[10,11].

**Cross-linking mass spectrometry**

Samples for cross-linking mass spectrometry were prepared in the same way as those for cryo-EM. The purified hSAGA complex was cross-linked by bis (sulfosuccinimidyl) suberate ($BS^3$) (Thermo Fisher) with 1:1 mass ratio at room temperature for 1 hour. 50 mM Tris-HCl pH 7.5 was used to terminate the reaction after incubation. Cross-linked complexes were precipitated with cooled acetone and were dried by using a Speedvac. The pellet was dissolved in 8 M Urea, 100 mM Tris-HCl pH 8.5, followed by TCEP reduction, iodoacetamide alkylation, and overnight trypsin digestion. Tryptic peptides were desalted with MonoSpin™ C18 column (GL Science, Tokyo, Japan) and analyzed by a home-made 30 cm-long pulled-tip analytical column (75 µm ID packed with ReproSil-Pur C18-AQ 1.9 µm resin, Dr. Maisch GmbH, Germany) coupled to an EASY-nLC 1200 nano HPLC (Thermo Scientific, San Jose, CA). The peptide mixture was separated by applying a 120-minutes step-wise gradient of 5-100% buffer B (80% acetonitrile (ACN) in 0.1% formic acid) at a flow rate of 300 nl/min. Peptides eluted from the LC column were directly electrosprayed into the mass spectrometer and analyzed in positive mode using a data-dependent top-20 acquisition methods. Peptides containing the isopeptide bonds were identified and evaluated using pLink2 software[12]. Protein-protein cross-links were filtered with 5%FDR and plotted using xVis[13].

**Supplementary References**


1 Kastner, B. *et al.* GraFix: sample preparation for single-particle electron cryomicroscopy. *Nat Methods* **5**, 53-55 (2008).

2 Zheng, S. Q. *et al.* MotionCor2: anisotropic correction of beam-induced motion for improved cryo-electron microscopy. *Nat Methods* **14**, 331-332 (2017).

3 Grant, T. & Grigorieff, N. Measuring the optimal exposure for single particle cryo-EM using a 2.6 A reconstruction of rotavirus VP6. *Elife* **4**, e06980 (2015).

4 Zhang, K. Gctf: Real-time CTF determination and correction. *J Struct Biol* **193**, 1-12 (2016).

5 Zivanov, J. *et al.* New tools for automated high-resolution cryo-EM structure determination in RELION-3. *Elife* **7** (2018).

6 Bai, X. C., Rajendra, E., Yang, G., Shi, Y. & Scheres, S. H. Sampling the conformational space of the catalytic subunit of human gamma-secretase. *Elife* **4** (2015).

7 Scheres, S. H. & Chen, S. Prevention of overfitting in cryo-EM structure determination. *Nat Methods* **9**, 853-854 (2012).

8 Kucukelbir, A., Sigworth, F. J. & Tagare, H. D. Quantifying the local resolution of cryo-EM density maps. *Nat Methods* **11**, 63-65 (2014).

9 Pettersen, E. F. *et al.* UCSF Chimera--a visualization system for exploratory research and analysis. *J Comput Chem* **25**, 1605-1612 (2004).

10 Adams, P. D. *et al.* PHENIX: a comprehensive Python-based system for macromolecular structure solution. *Acta Crystallogr D Biol Crystallogr* **66**, 213-221 (2010).

11 Emsley, P., Lohkamp, B., Scott, W. G. & Cowtan, K. Features and development of Coot. *Acta Crystallogr D Biol Crystallogr* **66**, 486-501 (2010).

12 Chen, Z. L. *et al.* A high-speed search engine pLink 2 with systematic evaluation for proteome-scale identification of cross-linked peptides. *Nat Commun* **10**, 3404 (2019).

13 Grimm, M., Zimniak, T., Kahraman, A. & Herzog, F. xVis: a web server for the schematic visualization and interpretation of crosslink-derived spatial restraints. *Nucleic Acids Res* **43**, W362-369 (2015).

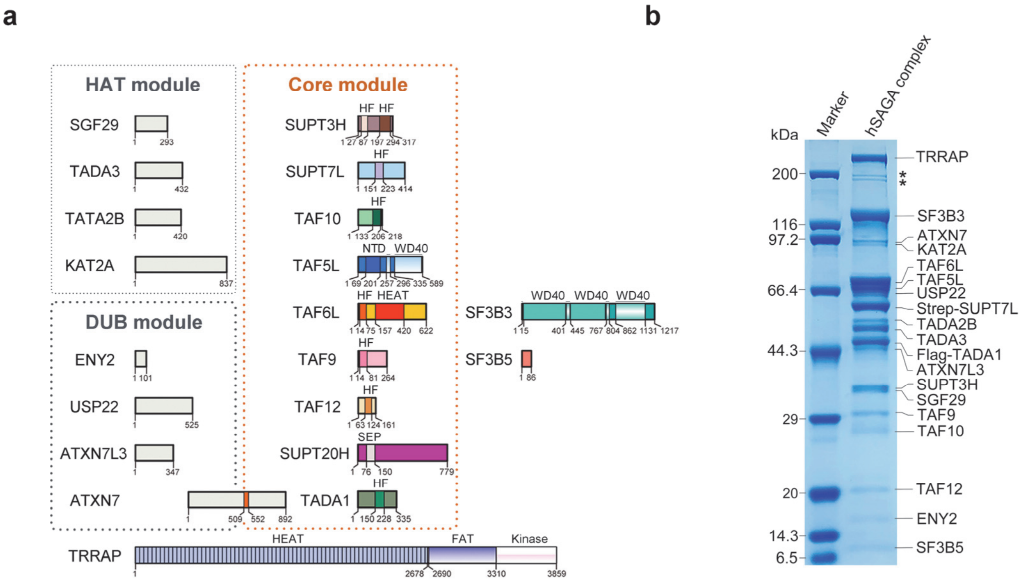


c

**Mass Spectra result for human SAGA complex**

| Subunit | Coverage | PSMs | Peptides | Unique peptides |
|---|---|---|---|---|
| TRRAP | 77 | 672 | 269 | 269 |
| SF3B3 | 71 | 315 | 75 | 75 |
| ATXN7 | 41 | 49 | 25 | 25 |
| KAT2A | 51 | 76 | 45 | 39 |
| SUPT20H | 59 | 86 | 37 | 37 |
| TAF6L | 75 | 152 | 44 | 44 |
| TAF5L | 79 | 129 | 39 | 39 |
| USP22 | 33 | 23 | 15 | 15 |
| SUPT7L | 77 | 61 | 21 | 21 |
| TADA2B | 66 | 50 | 27 | 27 |
| TADA3 | 74 | 80 | 34 | 34 |
| TADA1 | 60 | 82 | 21 | 21 |
| ATXN7L3 | 15 | 6 | 3 | 3 |
| SUPT3H | 48 | 39 | 12 | 12 |
| SGF29 | 63 | 39 | 20 | 20 |
| TAF9 | 78 | 59 | 19 | 10 |
| TAF10 | 38 | 25 | 7 | 7 |
| TAF12 | 40 | 19 | 8 | 8 |
| ENY2 | 39 | 9 | 5 | 5 |
| SF3B5 | 91 | 21 | 7 | 7 |

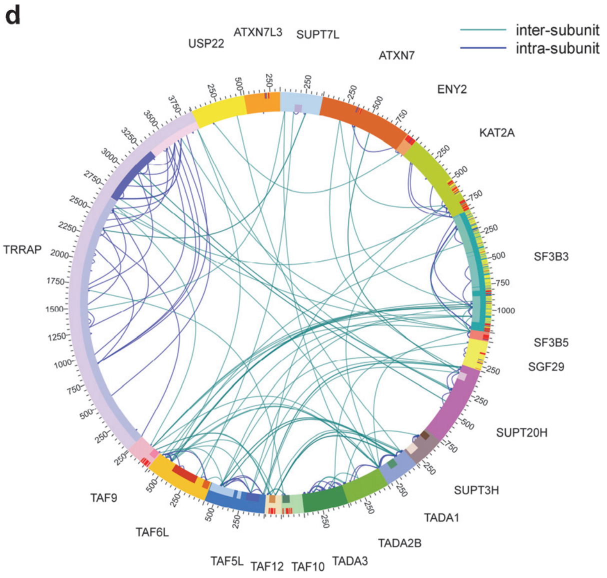


**Supplementary Fig. S1. Purification and characterization of endogenous human SAGA complex. a,** Schematic drawing of the domain organizations of hSAGA complex. Residues at domain boundaries are indicated. **b,** SDS-PAGE analysis of the endogenous hSAGA complex after tandem affinity purification; *asterisks* indicate nonspecific proteins. **c,** Mass spectrometry analysis of the purified hSAGA complex. PSMs stands for peptide spectrum matches, which were used to indicate the relative abundance of a certain protein. Unique peptides were used for protein identification. **d,** Overview of the cross-linking mass spectrometry data. High confidence lysine-lysine intra-subunit (blue) and inter-subunit (green) cross-links were denoted.

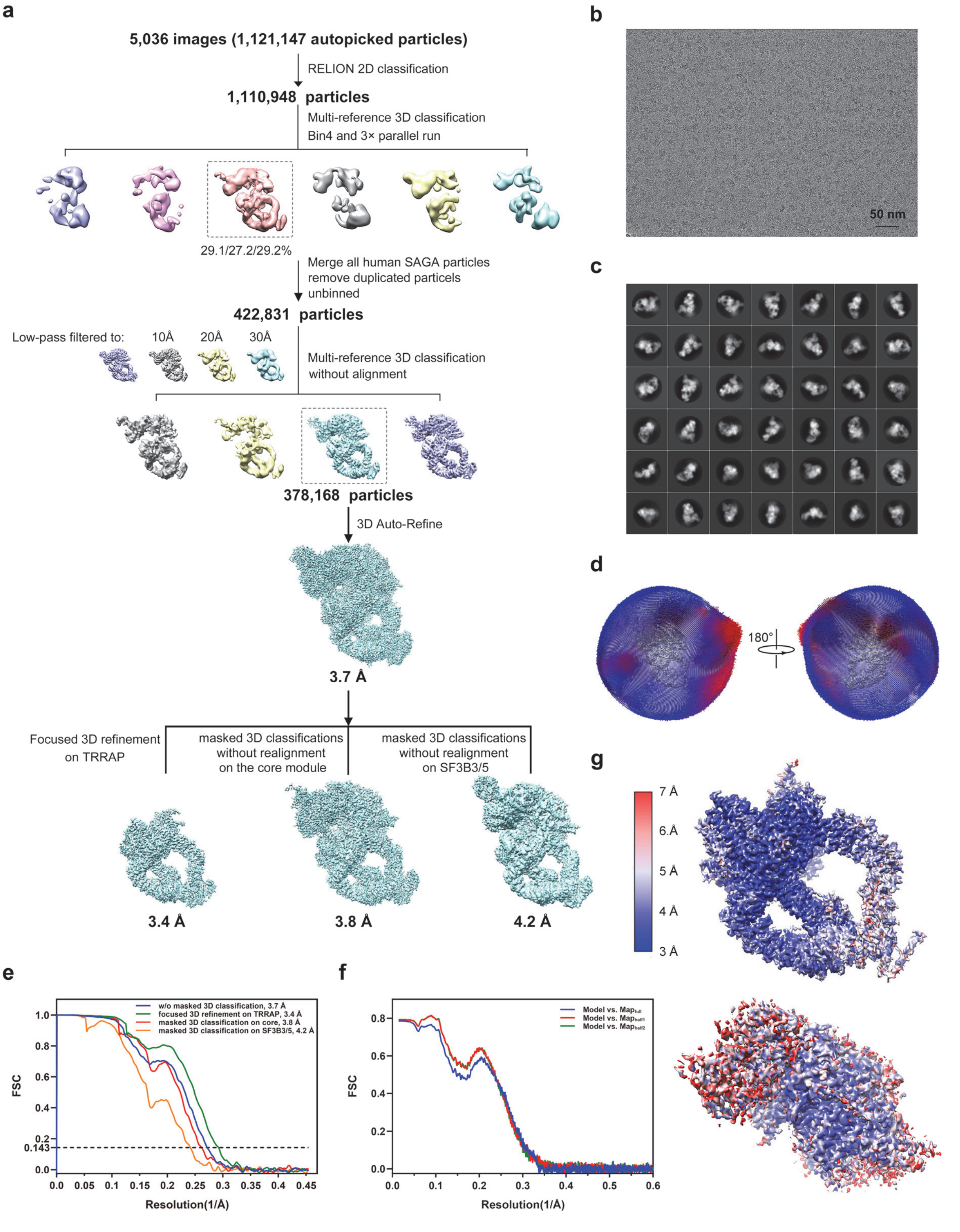


**Supplementary Fig. S2. Cryo-EM analysis of human SAGA complex. a,** A brief flow chart for the cryo-EM data processing of hSAGA complex. The final reconstruction has an overall

resolution of 3.7 Å. Focused refinement were applied to TRRAP (left branch), and masked 3D classifications without realignment were applied to core module (middle branch) and SF3B3/5 (right branch). More details are available in Methods. **b,** Representative micrograph of the cryo-EM dataset of hSAGA complex. **c,** Representative 2D class averages obtained from cryo-EM particles of the hSAGA complex. **d,** Angular distributions of particles in front and back views for the final reconstruction of hSAGA complex. **e,** Resolution assessment of the cryo-EM maps using Fourier shell correlation (FSC) at the 0.143 criterion. **f,** The map-model FSC curves for cross-validation: model versus full map (blue), model versus half map 1 (red), model versus half map 2 (green). The model was refined against half map 1 but not half map 2. The overall consistency between the red and green curves indicates that the refinement of the atomic coordinates did not suffer from severe over-fitting. **g,** Local-resolution estimates of TRRAP (top) and the core module (bottom) of hSAGA complex structure.

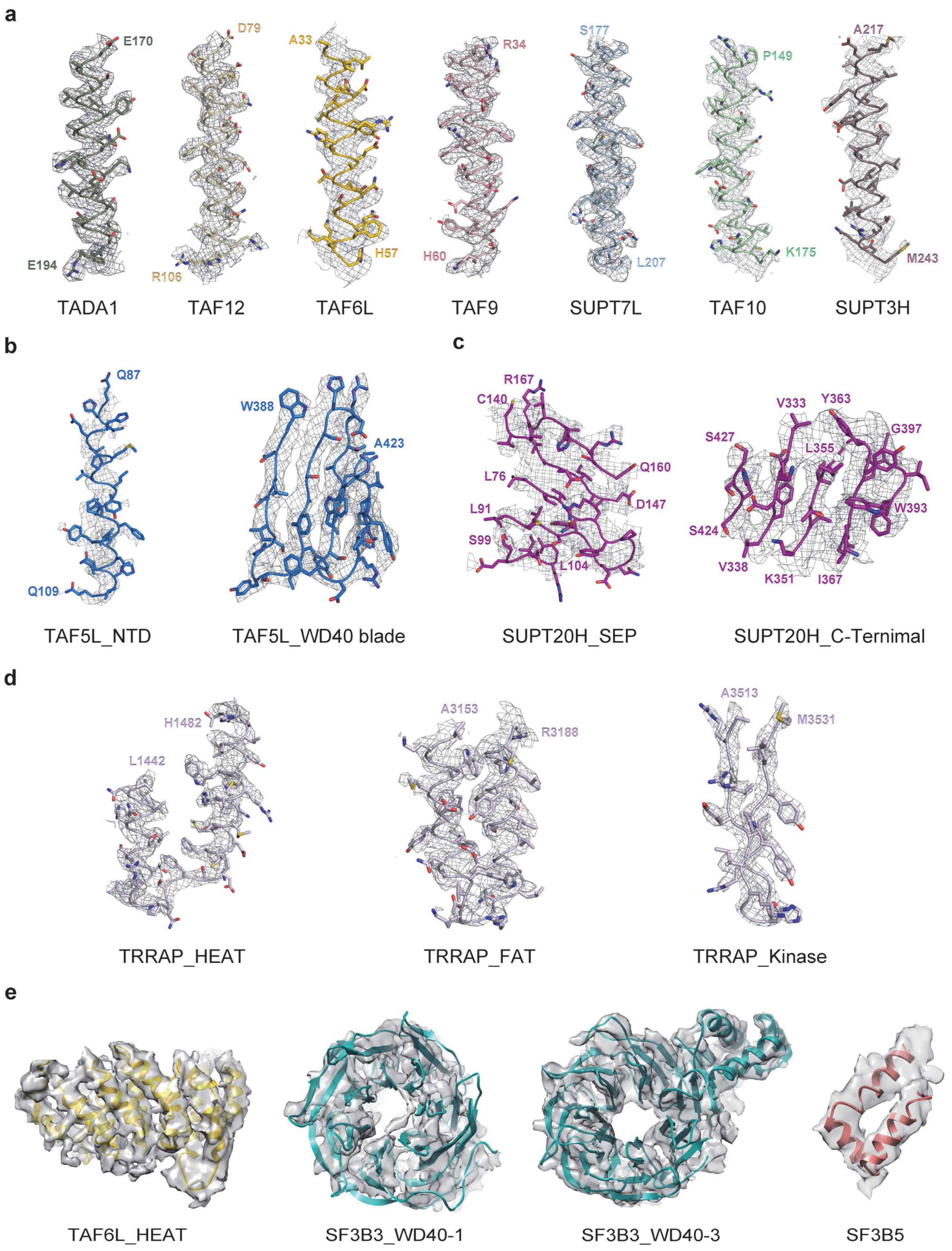


**Supplementary Fig. S3. Cryo-EM data quality of human SAGA complex.** Representative EM density maps for subunits of the hSAGA complex contoured at 0.009-0.015.

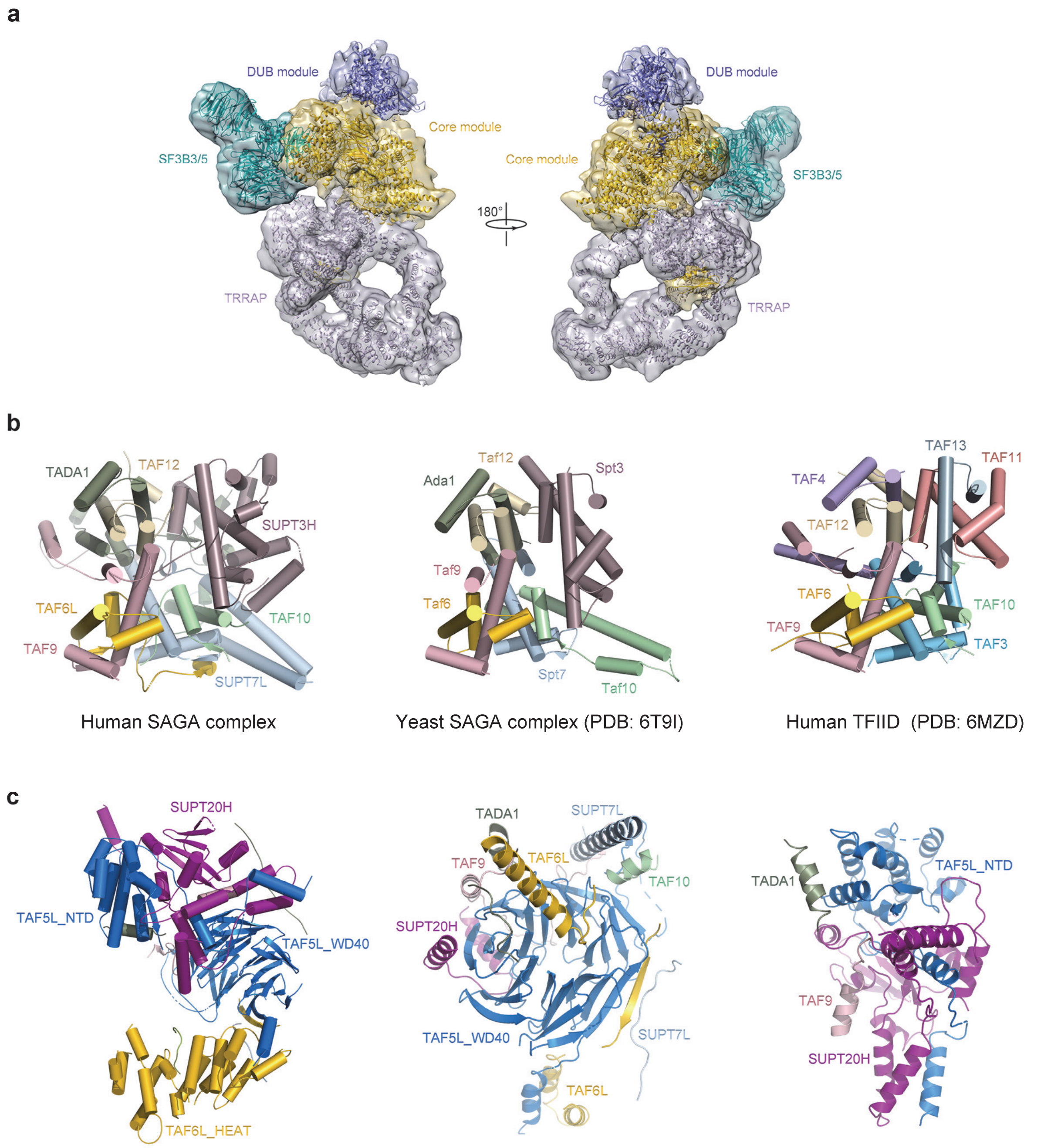


**Supplementary Fig. S4. Structural characterization of the core module of human SAGA complex. a,** A low-resolution cryo-EM density map (contoured at 0.001-0.0025) of hSAGA complex fitted with the atomic model of hSAGA, shown in front and back views. The core module structure of yeast SAGA complex was superposed with that of the hSAGA complex, leading to the fit of yeast DUB module exactly in the extra densities (colored in blue) of the low-resolution hSAGA map. **b,** Structural comparison of the histone octamer-like fold of hSAGA complex with that of yeast SAGA complex (PDB: 6T9I) and that of human TFIID complex (PDB: 6MZD). Homologous proteins are denoted with the same color. **c,** Left panel: overall structural organization of TAF5L, SUPT20H and the HEAT repeat domain of TAF6L; middle

panel: interaction interfaces between the WD40 domain of TAF5L and its adjacent hSAGA subunits; right panel: interaction interfaces among the N-terminal domain of TAF5L, SUPT20H, TADA1 and TAF9.

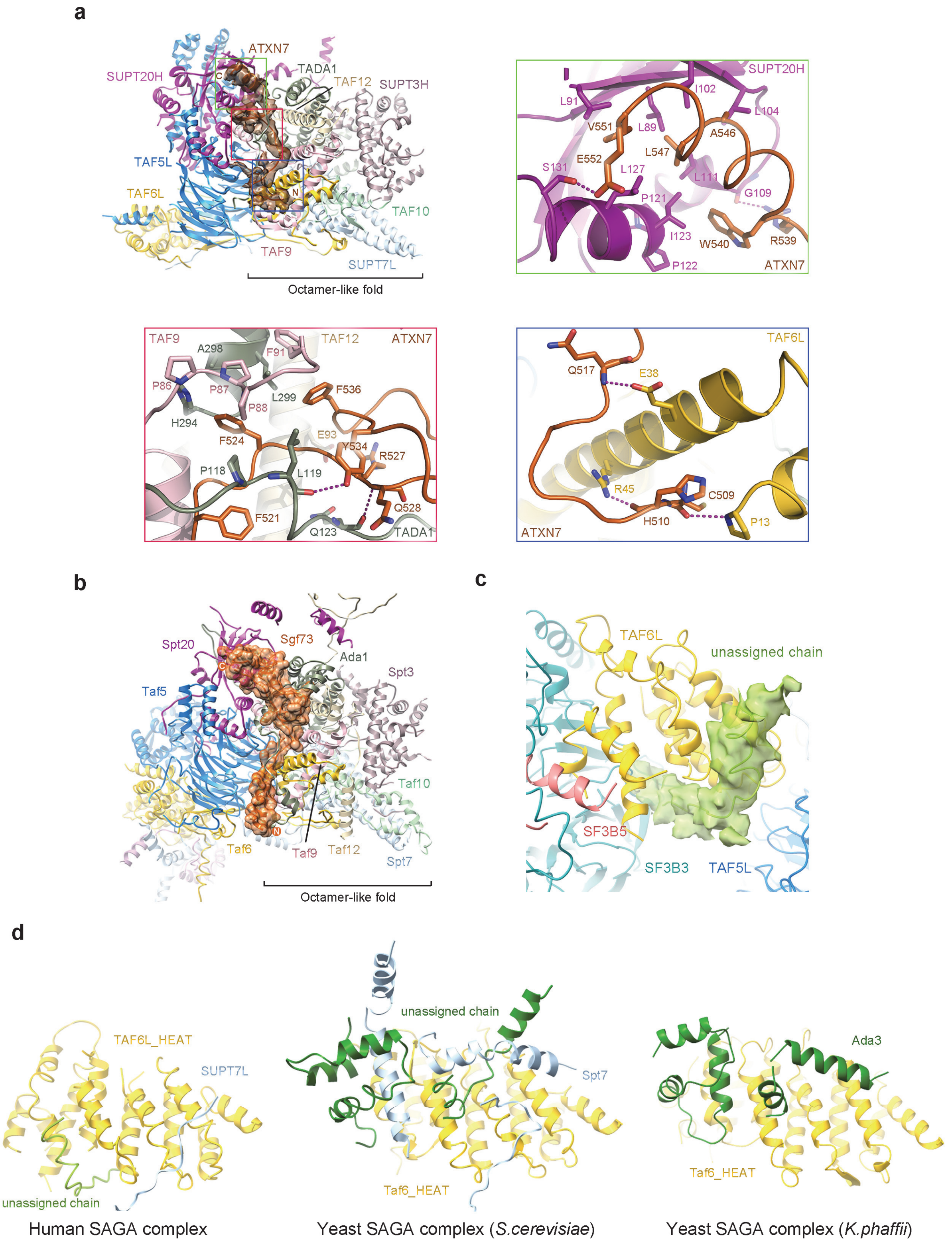


**Supplementary Fig. S5. Flexible tethering of the enzymatic modules of human SAGA complex. a,** Details interfaces between ATXN7 and the core module. Residues that mediate the interactions at the interface are shown in stick representation. Dashed magenta lines denote the

intermolecular hydrogen-bonding interactions. **b,** Residues 353-437 of Sgf73 meanders through the core module of the yeast SAGA (PBD: 6T9I). Yeast Sgf73 residues are in surface representation and the core module subunits of yeast SAGA are shown in cartoon. The color scheme is the same as their orthologous in hSAGA complex. **c,** Additional EM density (contoured at 0.009) passes through the gap between TAF6L and SF3B3 but could not be assigned. **d,** Comparison of the convex surface of the TAF6L HEAT repeats in hSAGA complex (left) and yeast SAGA complex (middle and right), showing that the unassigned chain on the surface of TAF6L occupies the similar position as Ada3 does in yeast SAGA complex (PDB: 6T9I and 6TB4).

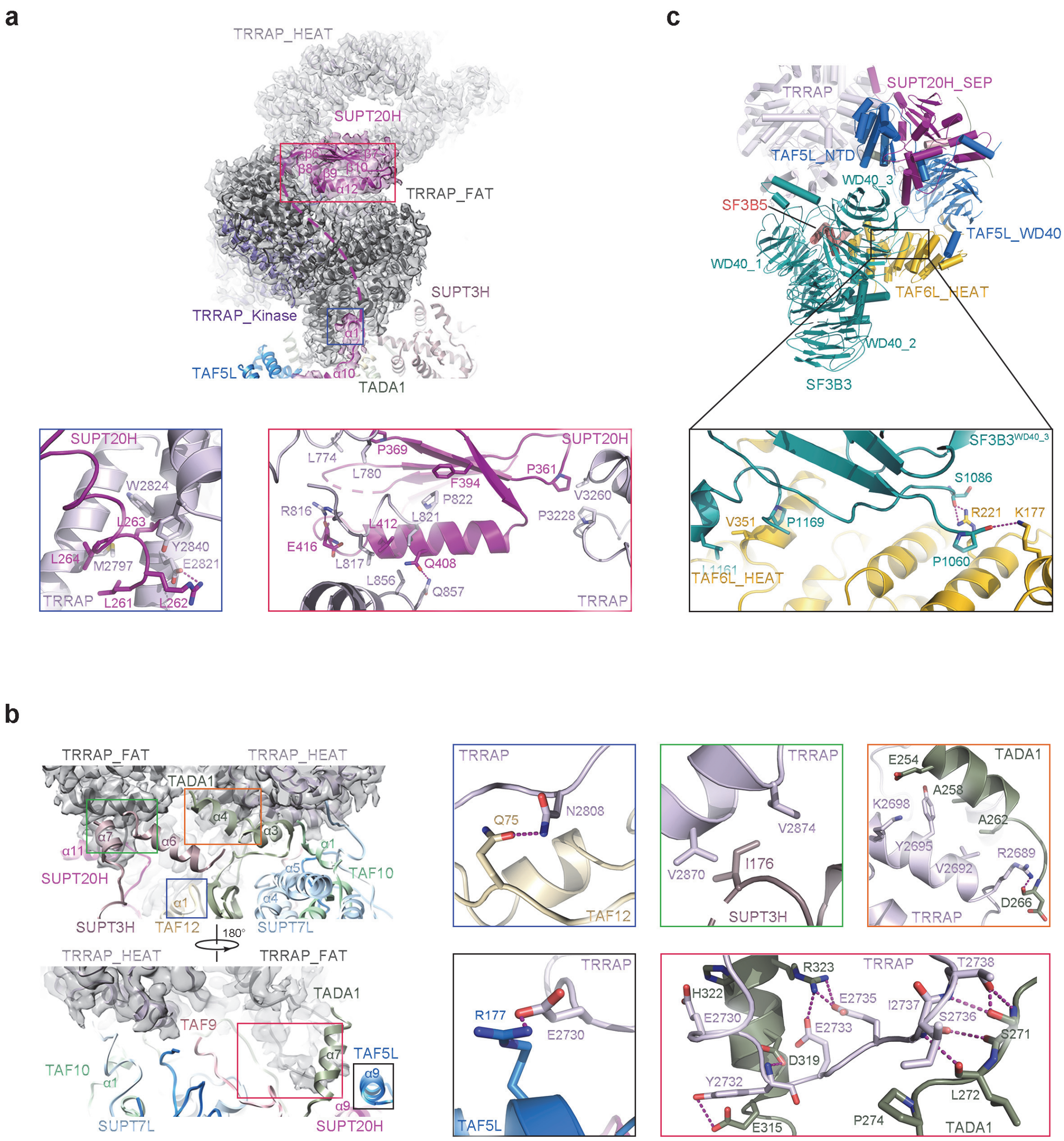


**Supplementary Fig. S6. Structural analyses of the interfaces among TRRAP, SF3B3/5 and the core module. a-b,** Close-up view of the contacts between TRRAP and the core module. **(a)**, Details of interactions between TRRAP and SUPT20H. **(b)**, Detailed view of the interface between TRRAP and the other core module components related to Fig. 1g. Residues that mediate the interactions at the interface are shown in stick representation. Dashed magenta lines denote the intermolecular hydrogen-bonding interactions. **c,** Close-up view of the contacts between SF3B3/5 and the core module.

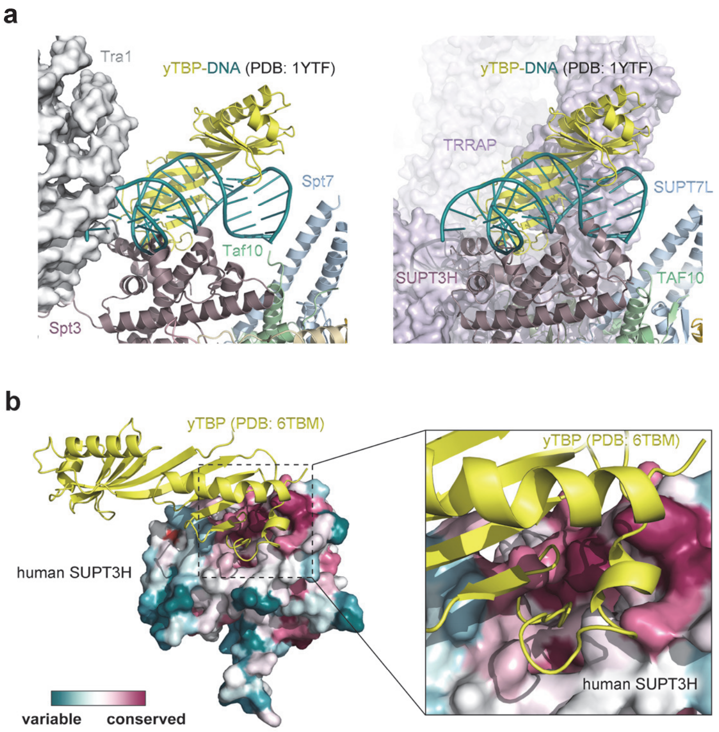


**Supplementary Fig. S7. Modeling of TBP into the human SAGA complex structure. a,** The TBP-DNA complex structure (PDB: 1YTF) was superposed with the yeast SAGA structure (left panel) and with the hSAGA structure modelled with TBP (right panel), respectively. **b,** The TBP structure (yellow) was modelled into the conserved TBP-binding pocket of SUPT3H by superposing the structures of yeast Spt3-TBP (PDB: 6TBM) and human SUPT3H (left panel). A close-up view of the TBP-binding pocket was shown in the right panel. SUPT3H is colored according to the degree of amino acid conservation among SUPT3H homologues (ruby, well conserved; teal, highly variable).

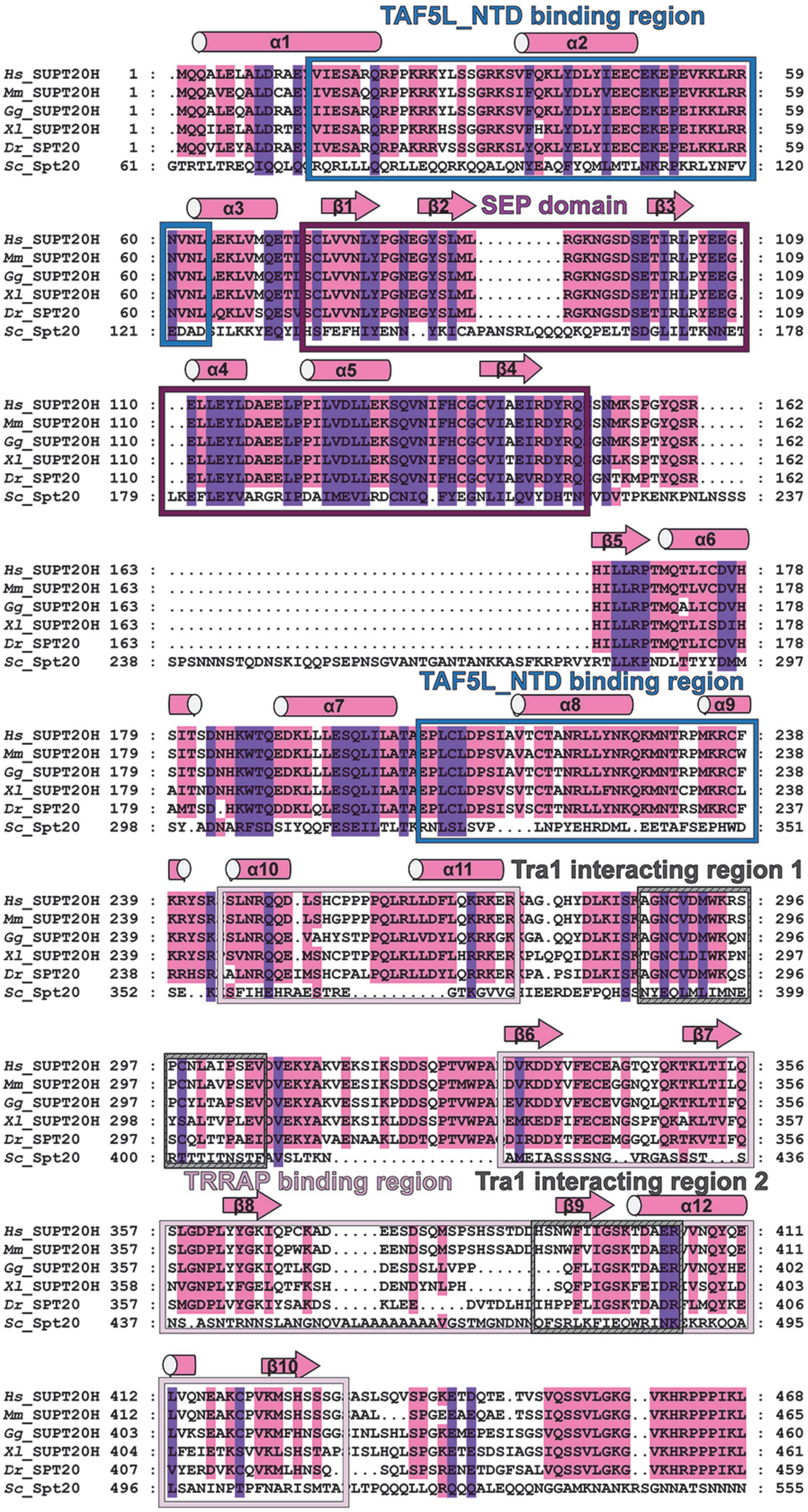
TAF5L_NTD binding region
α1
α2
Hs_SUPT20H
Mm_SUPT20H
Gg_SUPT20H
Xl_SUPT20H
Dr_SPT20
Sc_Spt20
α3
β1
β2
SEP domain
β3
α4
α5
β4
β5
α6
TAF5L_NTD binding region
α7
α8
α9
α10
α11
Tra1 interacting region 1
β6
β7
TRRAP binding region
Tra1 interacting region 2
β8
β9
α12
β10

**Supplementary Fig. S8. Sequence alignment of SUPT20H across several model organisms.** The TAF5L_NTD binding region, the conserved SEP domain, the two Tra1 interacting regions of the yeast Spt20, and the TRRAP binding region are denoted with blue, magenta, dark gray, and light pink boxes, respectively. Secondary structure assignments are shown as cylinders (α helices) and arrows (β strands), and conserved residues are highlighted in purple (100% of conservation) and pink (80% of conservation). The model organisms include *Homo sapiens* (*Hs*), *Mus musculus* (*Mm*), *Gallus gallus* (*Gg*), *Xenopus laevis* (*Xl*), *Danio rerio* (*Dr*), *Saccharomyces cerevisiae* (*Sc*).

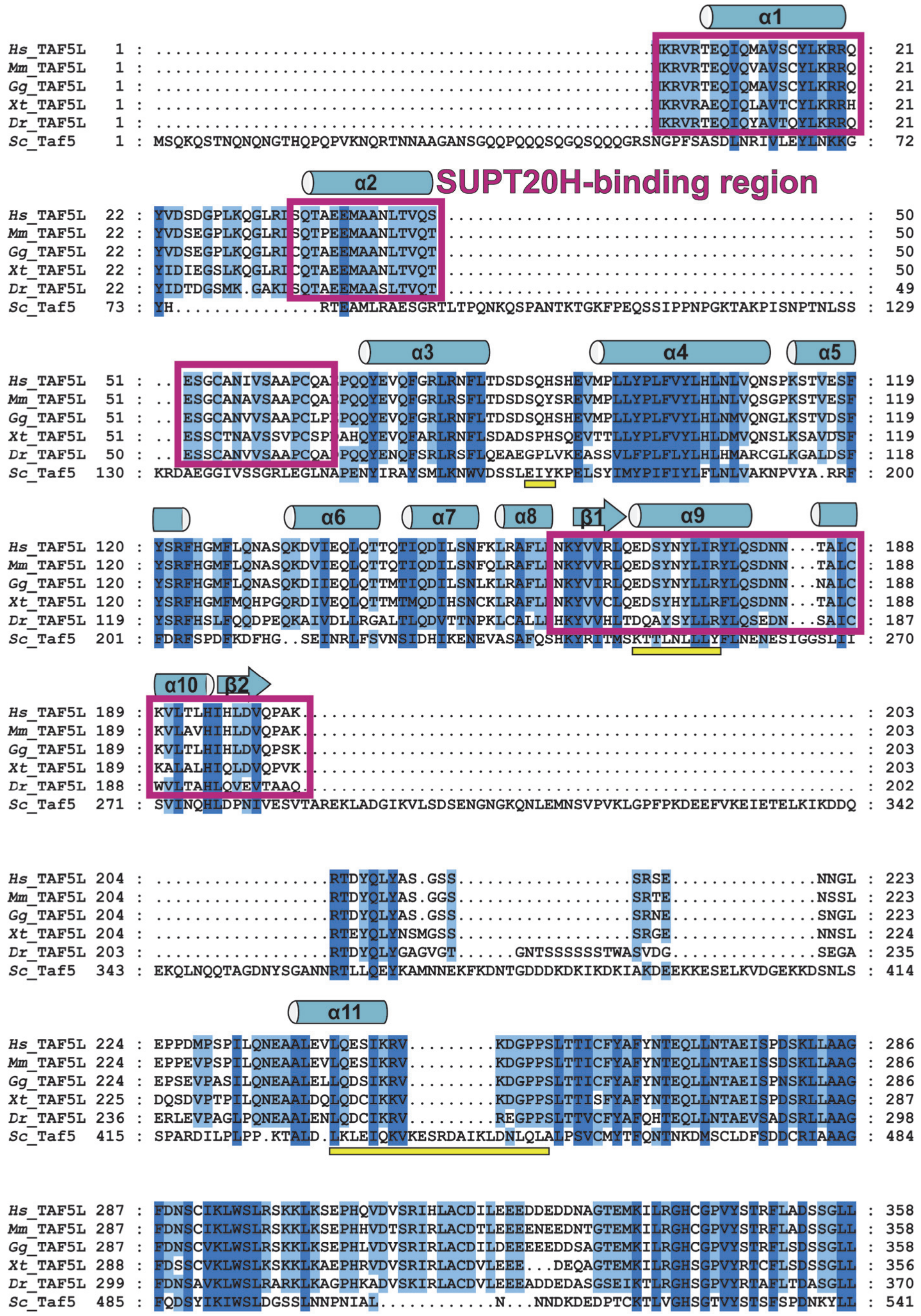


**Supplementary Fig. S9. Sequence alignment of the N-terminal domain of TAF5L orthologues with that of yeast Taf5.** The SUPT20H binding region of TAF5L is denoted with magenta boxes. The Taf6-interacting region in yeast Taf5 is underlined with yellow color. Secondary structure assignments are shown as cylinders (α helices) and arrows (β strands), and conserved residues are highlighted in blue (100% of conservation) and light blue (80% of conservation). The model organisms include *Homo sapiens* (*Hs*), *Mus musculus* (*Mm*), *Gallus gallus* (*Gg*), *Xenopus tropicalis* (*Xt*), *Danio rerio* (*Dr*), *Saccharomyces cerevisiae* (*Sc*).

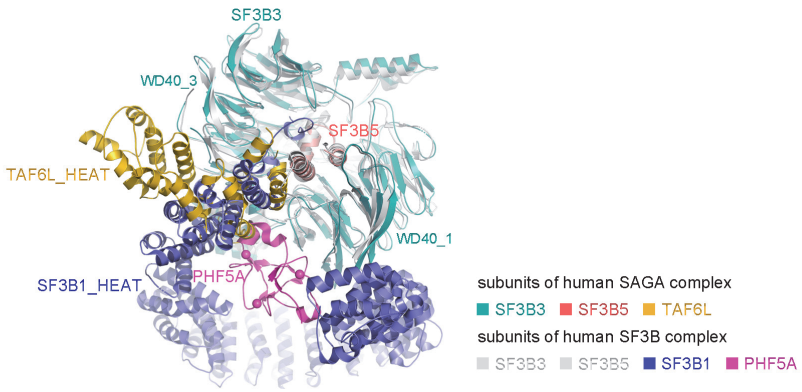


**Supplementary Fig. S10. Structural comparison of the incorporation of SF3B3 and SF3B5 into the human SAGA complex and the SF3B complex.** The SF3B3-SF3B5-TAF6L_HEAT structure from the human SAGA complex is superposed with the SF3B complex structure (PDB: 5IFE). SF3B3 and SF3B5 are shared subunits in the two complexes.

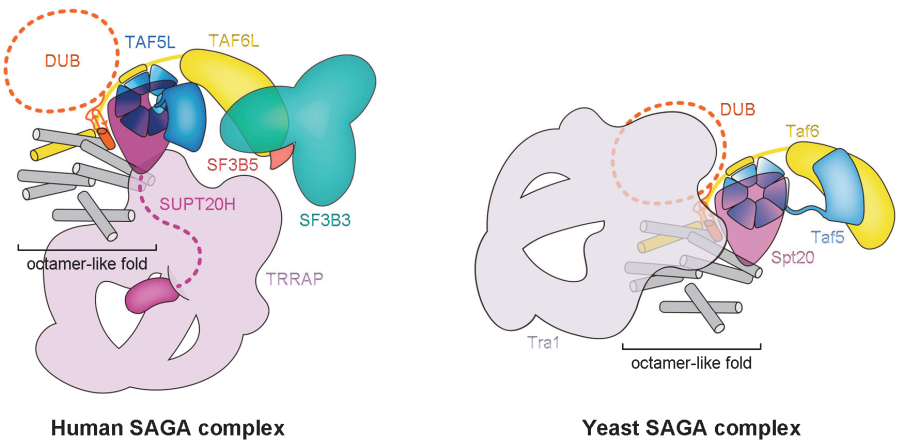


**Supplementary Fig. S11. Cartoon illustration of the major differences in the structural organization of human SAGA and yeast SAGA complexes.**

**Supplementary Table S1. Cryo-EM Data Collection and Refinement Statistics**

| | **Human SAGA complex** |
|---|---|
| **Data collection and processing** | |
| Microscope | Titan Krios |
| Detector | K3 |
| Voltage (kV) | 300 |
| Electron exposure (e-/ $Å^2$) | 50 |
| Defocus range (µm) | -0.8 to -3.0 |
| Pixel size (Å) | 1.10 |
| Symmetry imposed | C1 |
| Initial particle images (no.) | 1,121,147 |
| Final particle images (no.) | 378,168 |
| Map resolution (Å) | 3.70 |
| FSC threshold: 0.143 | |
| | |
| **Refinement** | |
| Initial model used (PDB code) | 6MZD, 6T9I and 6TBM |
| Map sharpening B factor ($Å^2$) | -108 |
| Model composition | |
| Non-hydrogen atoms | 52,583 |
| Protein residues | 6,589 |
| R.m.s. deviations | |
| Bond lengths (Å) | 0.003 |
| Bond angles (°) | 0.803 |
| Validation | |
| MolProbity score | 1.56 |
| Clashscore | 3.26 |
| Poor rotamers (%) | 0.05 |
| Ramachandran plot | |
| Favored (%) | 93.19 |
| Allowed (%) | 6.81 |
| Disallowed (%) | 0.00 |

**Supplementary Table S2. Summary of Model Building for the Human SAGA Complex**

| Subunit | Chain ID | Total residues | Modelled residues | Template PDB ID | Modelling |
|---|---|---|---|---|---|
| SF3B3 | A | 1217 | 1-379;384-645;662-691;695-829;834-1064;1086-1216 | 5IFE | rigid fitting |
| SF3B5 | B | 86 | 31-75 | 5IFE | rigid fitting and manually built |
| TRRAP | C | 3859 | 19-34;38-119;128-173;223-238;242-259;277-298;306-467;541-566;578-606;625-774;778-1221;1226-1483;1509-1616;1635-1679;1689-1741;1748-1784;1803-1843;1857-1953;1960-1971;1979-1994;2001-2020;2090-2108;2122-2133;2142-2155;2162-2224;2236-2255;2265-2307;2316-2377;2387-2427;2435-2528;2586-3008;3025-3089;3096-3283;3293-3379;3415-3786;3803-3859 | 6T9I 6TBM | homology modelling |
| SUPT20H | D | 779 | 2-24;35-91;99-150;158-273;332-339;347-369;391-429 | 6T9I 6TBM | homology modelling |
| SUPT3H | E | 317 | 26-107;159-176;180-245;280-315 | 6T9I 6TBM | homology modelling |
| TADA1 | G | 335 | 108-127;138-228;251-327 | 6T9I 6TBM | homology modelling |
| TAF5L | H | 589 | 2-24;34-126;133-201;235-250;253-323;331-540;552-583 | 6T9I 6TBM | homology modelling |
| SUPT7L | I | 414 | 22-29;61-87;129-261 | 6T9I 6TBM | homology modelling |
| TAF6L | K | 622 | 13-93;100-134;145-248;260-370; | 6T9I 6TBM | homology modelling |
| TAF9 | M | 264 | 13-131 | 6MZD | rigid fitting and manually built |
| TAF10 | O | 218 | 116-180;192-217 | 6MZD | rigid fitting and manually built |
| TAF12 | R | 151 | 59-130 | 6MZD | rigid fitting and manually built |
| ATXN7 | L | 892 | 509-552 | | De novo building |
| unassigned | X | | 1-19 | | Built with Ala |
